# Effects of Rotary and Deep Tillage on Soil Environment and Melon Root Development

**DOI:** 10.3390/plants13182611

**Published:** 2024-09-19

**Authors:** Miao Yan, Tao Xiong, Juntao Yang, Ting Wu, Jiancai Mao, Xiaotian Tang, Guozhi Hu

**Affiliations:** Hami Melon Research Center, Xinjiang Academy of Agricultural Sciences, Urumqi 830091, China; yanmiao0901@126.com (M.Y.);

**Keywords:** melon, tillage practices, soil nutrients, soil microorganisms, root development

## Abstract

Tillage practices significantly influence crop yield and soil quality. This study investigated the impact of rotary tillage (RT) and deep tillage (DT) on soil properties, microbial diversity, and melon (*Cucumis melo* L.) root growth and yield. RT involved breaking up the topsoil to a depth of 15 cm using a rotary tiller, while DT employed a rotary tiller followed by a moldboard plow to turn the soil layer over to a depth of 35 cm. The melon variety “Nasimi” was used as the material. Our findings revealed a remarkable response of soil phosphorus to tillage practices. High-throughput sequencing results revealed a significant impact of tillage practices on the soil fungal composition, richness, and diversity but little impact on the bacterial communities. Compared to RT, DT markedly enhanced melon root length, root surface area, root volume, and mean root diameter by 47.42%, 56.70%, 58.83%, and 27.28%, respectively. Additionally, DT treatments significantly increased melon yield (53.46%) compared to RT. The results indicate that DT improves soil nutrient availability, affects soil fungal community characteristics, and optimizes root distribution in soil, thereby improving melon yield. The findings offer valuable theoretical insights for the implementation of effective tillage practices in open-field melon cultivation.

## 1. Introduction

Xinjiang Province, situated in the Eurasian hinterland, exhibits a unique climate characterized by pronounced diurnal temperature fluctuations, arid conditions, and extensive sunlight exposure. These climatic conditions have fostered a favorable environment for melon and fruit cultivation [1]. Cultivation techniques, particularly tillage practices, significantly influence crop yield and soil quality. However, the field of open-field melon (*Cucumis melo* L.) and vegetable tillage in China remains relatively underdeveloped [2]. Rotary tillage currently dominates the sector in Xinjiang. While rotary tillage is widely adopted due to its convenience and low cost [3], its continuous use has led to a series of detrimental consequences. These include shallow topsoil layers, compacted subsoil, and nutrient accumulation in the topsoil [4]. Deep tillage breaks through compacted subsoil layers, enhancing nutrient availability and root development [5]. However, it may also lead to increased nitrogen loss [6]. Conservation tillage minimizes soil disturbance by retaining crop residues on the soil surface, thereby reducing erosion and conserving energy and resources [7,8]. This practice was adopted in greenhouse melon (*Cucumis melo* L.) cultivation [9]. However, due to the extreme drought in the Turpan region of Xinjiang, conservation tillage practices are not conducive to crop root development. Therefore, optimizing tillage practices to improve soil structure, activate soil nutrients, and increase resource utilization efficiency plays a pivotal role in enhancing soil fertility and boosting crop yields [10,11]. Deep tillage has been identified as a successful strategy for enhancing soil properties and optimizing nutrient utilization [12].

Soil enzymes, secreted by soil microorganisms, flora, and fauna, are biologically active substances with remarkable catalytic abilities. They respond rapidly and accurately to subtle soil changes [13,14], making them valuable early indicators of soil quality alterations induced by shifts in agricultural tillage practices. These changes, often difficult to detect, are typically characterized by utilizing the sensitivity and ease of measurement of soil enzymes. Soil microorganisms constitute the most crucial active component of the soil ecosystem, governing critical processes such as the decomposition and formation of soil organic matter, nutrient cycling, and energy flow. A rich microbial community is essential for an ecosystem [15,16]. However, soil microbial communities are susceptible to a range of factors, including tillage practices, climate, and fertilization. Tillage practices, in particular, can disrupt soil physicochemical properties and enzyme activity, leading to disturbances in soil microbial diversity [17,18,19]. Consequently, the impact of tillage practices on soil microbial diversity has garnered significant attention [20].

The root system, essential for crops to absorb water and nutrients, and synthesize various physiologically active substances, plays a pivotal role in crop growth and yield [21,22]. Plant root development is influenced by the heterogeneous distribution of mineral nutrients and water in the soil. In turn, plants can adjust root development to acquire soil water and nutrients, directly impacting their adaptability to the environment and final yield [23,24]. Among root morphological parameters, root length and root surface area are closely related to the plant’s ability to absorb soil nutrients and water. Longer roots and a larger root surface area enhance the roots’ capacity to absorb nutrients and extend their reach, enabling them to firmly grasp deeper soil layers. Root mean diameter, root tip count, and other indicators are crucial parameters for assessing root spatial distribution and reflect the roots’ ability to absorb and exchange substances in the soil. Fractal dimension, typically ranging from 1 to 2, represents the complexity of root branching. A higher fractal dimension indicates a more intricate root system and a higher degree of root development [25,26]. The impact of different tillage practices on crop root structure and shape has been documented in various crops, including soybean [27], maize [28], and pea [29]. However, studies on melon are scarce. Developing optimal tillage practices to enhance root system development and subsequent water and nutrient acquisition is crucial for maximizing melon yield and stability.

In agricultural practices, deep tillage promotes root development and enhances nutrient absorption capacity, which is crucial for high and stable melon yields. In response to the pressing issues of soil degradation, nutrient loss, and yield decline in melon cultivation. A comparative study was conducted on rotary and deep tillage to investigate their impact on soil properties, microbial diversity, root growth, and subsequent crop performance in melon cultivation. The objective was to identify optimal tillage practices for establishing a fertile soil profile and maximizing melon yield and efficiency.

## 2. Results

### 2.1. Impact of Tillage Practices on Soil Chemical Properties

Soil pH is a crucial indicator of soil physicochemical properties, significantly influencing soil nutrient availability. In this study, soil pH in all treatments and soil layers remained close to neutral (6.5 < pH < 7.5). Deep tillage (DT) significantly reduced soil pH in the 0–10 cm soil layers. Soil total phosphorus (TP) and available phosphorus (AP) contents were highly responsive to tillage practices. Both TP and AP contents were significantly elevated in all two soil layers (0–10 and 10–20 cm) under DT compared to RT. TP content increased by 83.01% and 151%. AP content increased by 27.67% and 186%. Soil total nitrogen (TN), available potassium (AK), and total potassium (TK) contents were significantly increased by 16.46%, 22.09%, and 20.32% only in the 0–10 cm soil layers under DT (Table 1).

### 2.2. Impact of Tillage Practices on Soil Enzyme Activity

**Figure 1:**
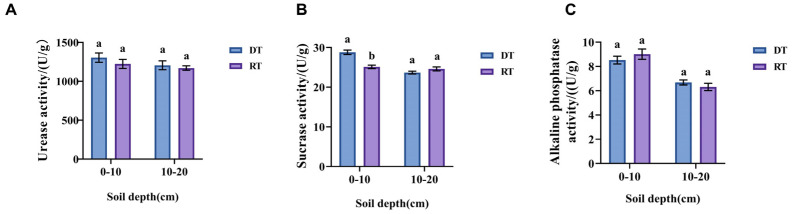
Changes in soil enzyme activity under different tillage practices. Different letters in RT (0–10) vs. DT (0–10) and RT (10–20) vs. DT (10–20) indicate significant differences (*p* < 0.05) according to an independent sample *t*-test. (**A**) Urease activity; (**B**) Sucrase activity; (**C**) Alkaline phosphatase activity. RT, rotary tillage; DT, deep tillage.

To assess the impact of tillage practices on soil enzyme activity, we investigated the activity levels of urease, sucrase, and alkaline phosphatase. Within the 0–20 cm soil layer, all these soil enzyme activities exhibited a gradual decrease with increasing soil depth. Soil urease and alkaline phosphatase activities exhibited no significant variation between tillage treatments (Figure 1A,C). DT treatment significantly enhanced soil sucrase activity in the 0–10 cm layer, with increases of 14.64% (Figure 1B).

### 2.3. Soil Microbial Diversity under Different Tillage Practices

**Figure 2:**
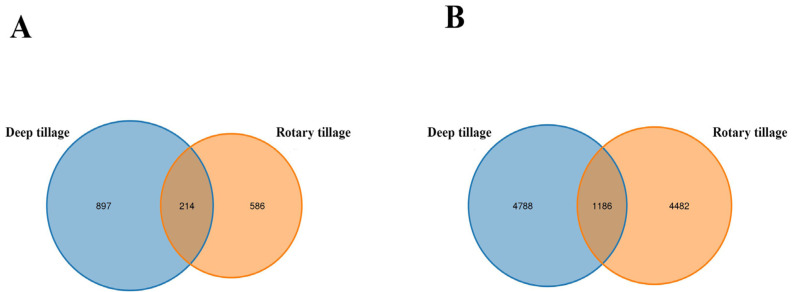
Venn diagrams of soil microbial OTUs under different tillage practices. Note: (**A**) OTU Venn diagram of fungi in soil; (**B**) OTU Venn diagram of bacteria in soil.

To assess the impact of tillage practices on soil microbial communities, we investigated the microbial diversity and composition. The fungal operational taxonomic units (OTUs) were determined for soil samples under different tillage treatments (Figure 2A). A total of 1453 OTUs were detected across all samples, with a shared number of 214. The number of unique fungal OTUs under DT treatment was 897, higher than the 586 under RT treatment. An analysis of bacterial OTUs in soil samples revealed a total of 9270 OTUs, with a shared number of 1186. The number of OTUs for DT and RT treatments was similar, at 4788 and 4482, respectively (Figure 2B). These findings suggest that DT treatment had a more significant impact on the number of fungal OTUs.

We further determined the alpha diversity of soil bacterial and fungal communities (Table 2). The ACE (Abundance-based Coverage Estimator) and Chao1 indices are commonly used to represent species richness, with their values positively correlated with the level of species richness. The Shannon index, on the other hand, is used to represent species diversity, with higher values indicating greater diversity in the sample. As shown in Table 2, both fungal diversity and richness were higher under DT than under RT treatment. DT treatment only increased bacterial diversity, and the differences between the indices were not significant. These results suggest that DT had a more pronounced effect on the richness and diversity of the fungal community.

**Figure 3:**
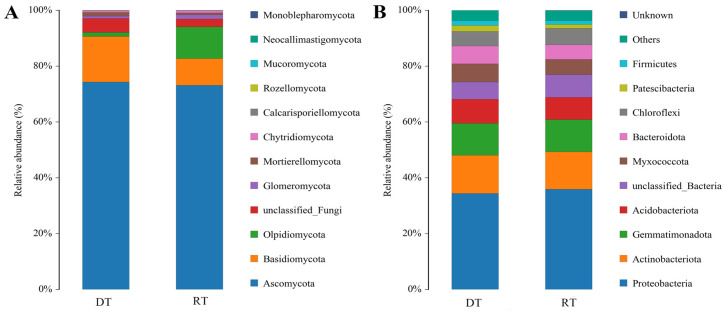
Comparison of soil microbial compositions under different tillage practices at the phylum level. (**A**) Fungi; (**B**) bacteria. RT, rotary tillage; DT, deep tillage.

At the phylum level, sequencing analysis revealed the presence of the following fungal phyla in the samples: Ascomycota, Basidiomycota, Olpidiomycota, and Glomeromycota (Figure 3A). These four phyla were the dominant phyla (relative abundance > 1%). Among all samples, Ascomycota had the highest relative abundance, with average values of 74.37% and 72.98% under DT and RT treatments, respectively. Compared to the RT treatment, DT treatment increased the relative abundance of Basidiomycota. The bacterial phyla detected in the samples are shown in Figure 3B. The dominant phyla included Proteobacteria, Actinobacteriota, Gemmatimonadota, Acidobacteriota, Myxococcota, Bacteroidota, Chloroflexi, Patescibacteria, and Firmicutes. Among these, Proteobacteria had the highest relative abundance, with average values of 34.42% and 35.94% under DT and RT treatments, respectively.

**Figure 4:**
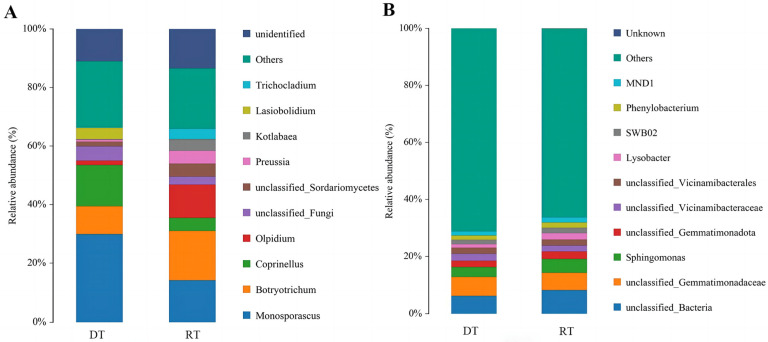
Comparison of soil microbial composition under different tillage practices at the genus level. (**A**) Fungi; (**B**) bacteria. RT, rotary tillage; DT, deep tillage.

As displayed in Figure 4A, fungal community analysis revealed Ascomycota as the dominant phylum, with Monosporascus as the most abundant genus (29.91% and 14.17% relative abundance under DT and RT, respectively). Preussia abundance increased significantly under RT. Proteobacteria, including Lysobacter, MND1, Phenylobacterium, and Sphingomonas, predominated in the bacterial community, with no significant differences observed between treatments (Figure 4B). Our findings suggest a significant impact of tillage practices on the soil fungal composition, but little impact on the bacterial one in soil.

### 2.4. Redundancy Analysis of Soil Microbial Communities and Soil Environmental Factors

**Figure 5:**
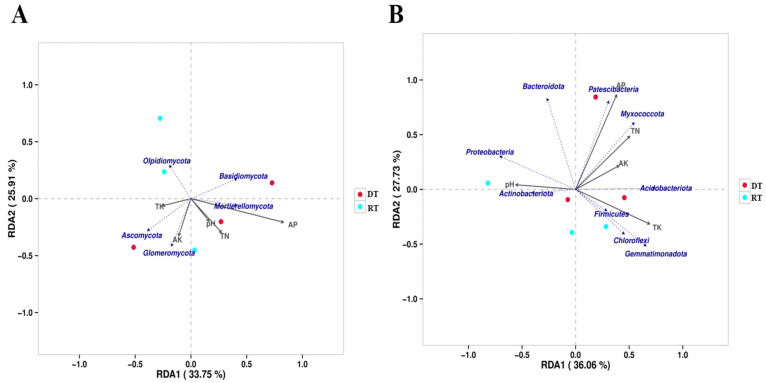
Redundancy analysis (RDA) of soil chemical properties and soil microbial composition under different tillage practices. (**A**) Fungi; (**B**) bacteria. Note: TN, total nitrogen; TK, total potassium; AP, available phosphorus; AK, available potassium. RT, rotary tillage; DT, deep tillage.

Redundancy analysis (RDA) was employed to investigate the influence of soil chemical properties on soil microbial communities (only dominant phyla are shown). For the soil fungal community, the first RDA axis explained 33.75%, the second axis explained 25.91%, and together they explained 59.66% of the variation in the soil fungal community. The phylum with the highest abundance, Ascomycota, was only negatively correlated with AP content. The magnitude of the influence of soil chemical properties on the fungal community structure was in the order AP > TN >AK > TK > pH (Figure 5A). For the soil bacterial community, the first RDA axis explained 36.06%, the second axis explained 27.73%, and together they explained 63.79% of the variation in the soil bacterial community. The phylum with the highest abundance, Proteobacteria, was only positively correlated with pH. The magnitude of the influence of soil chemical properties on the fungal community structure was in the order AP > TK > TN > pH > AK (Figure 5B). These findings suggest that AP content is the most important environmental factor simultaneously affecting both soil fungal and bacterial communities.

### 2.5. Effect of Different Tillage Practices on Melon Root Development

To assess the impact of different tillage practices on root development, we investigated various root morphological parameters. Morphological analysis revealed that under DT treatment, melon primary roots were more prominent and root density was higher, while under RT treatment, root hairs were more developed (Supplementary Figure S1). Parameter analysis showed that under DT treatment, melon root length, root surface area, root volume, and average root diameter were all significantly higher than under RT treatment, increasing by 47.42%, 56.70%, 58.83%, and 27.28%, respectively. The number of root tips also showed a significant increase of 23.89%. Fractal dimension showed a slight increase, with fractal dimensions of 1.59 and 1.62 under RT and DT treatments, respectively (Table 3). These results suggest that DT treatment positively affects melon root development.

### 2.6. Effect of Different Tillage Practices on Melon Yield

The effects of different tillage practices on melon yield are shown in Table 4. DT treatments significantly increased both weight per fruit (54.54%) and total yield (53.46%) compared to RT. While fruit shape index, an important melon quality trait, ranged from 1.30 to 1.40 across treatments without significant differences, DT demonstrated a clear yield advantage.

## 3. Discussion

Rational tillage practices are widely recognized as a key determinant of crop yield enhancement [30,31]. In this study, it was found that DT treatments significantly increased mean fruit weight and total melon yield compared to RT, aligning with previous research on open-field watermelon cultivation [32]. However, previous studies have indicated potential yield advantages of conservation tillage for watermelon [33] and melon [10]. This discrepancy suggests that environmental factors, such as soil texture and precipitation, play a crucial role in determining the optimal tillage practice.

Our findings also demonstrated that soil phosphorus responded remarkably to tillage practices. DT treatment significantly increased the content of total phosphorus and available phosphorus in the 0–20 cm soil layer. Phosphorus, as a major low-mobility element in soil, plays a critical role in regulating root development [34]. The root system is pivotal for plants, allowing them to absorb, transport, and utilize soil nutrients and water [35]. Roots exhibit developmental plasticity, and their function is closely related to root morphology and physiological characteristics. Plants often adapt to environmental changes by modifying root architecture and spatiotemporal distribution [36]. Studies on *Arabidopsis* have shown that root growth is inhibited under low-phosphorus conditions, while lateral root elongation is stimulated [37]. Other research suggests that plants can adapt to low-phosphorus environments by altering root architecture and enhancing root hair development [38]. Our results corroborate these findings, indicating that DT treatment optimized root distribution in the soil and had a more positive effect on melon root morphology and architecture. This could be attributed to two primary reasons: (1) Deep tillage promoted phosphorus mobility and enhanced phosphorus utilization, consequently influencing melon root development. (2) Rational deep tillage can break through the compacted barrier, creating a relatively loose and uniform 0–35 cm tillage layer, facilitating root growth and development [39,40].

Variations in soil environment and nutrient composition under different tillage practices can affect soil enzyme activity. Urease facilitates the hydrolysis of ammonium nitrogen, and its activity influences the conversion of organic nitrogen into readily available nitrogen. Sucrase, an enzyme involved in the soil organic carbon cycle, hydrolyzes sucrose into glucose and fructose, serving to some extent as an indicator of soil fertility and organic matter content [41,42]. In this study, we measured soil urease, sucrase, and alkaline phosphatase activity under different tillage treatments. The results revealed that soil urease and alkaline phosphatase activities exhibited no significant variation between tillage treatments. DT treatment significantly enhanced soil sucrase activity in the 0–10 cm layer, with increases of 14.64%. The activity of all three soil enzymes exhibited a gradual decrease with increasing soil depth, aligning with previous research demonstrating that topsoil enzyme activity is typically higher than subsoil enzyme activity [43,44]. This can be attributed to the fact that the topsoil is richer in organic matter compared to the subsoil. This abundance of organic matter provides ample substrates for microbial growth and metabolism, leading to the secretion of more soil enzymes by these microorganisms [45].

Tillage practices significantly influence soil fungal richness (ACE and Chao1 indices) and diversity (Shannon index). This study aligns with previous research, demonstrating that tillage practices markedly affect soil fungal richness and diversity [46,47]. However, soil bacterial richness and diversity exhibited no correlation with tillage practices. The observed disparity in the responses of fungal and bacterial communities to tillage practices can be attributed to several factors. Fungi, with their larger and more complex hyphal structures, are more susceptible to physical disturbances caused by tillage [48]. Additionally, fungi are generally more sensitive to environmental changes [19]. Hence, their community structure and function can serve as valuable indicators of soil health and resilience [49]. Understanding the differential responses of fungal and bacterial communities to tillage practices can inform the development of sustainable agricultural practices that optimize soil microbial diversity and function, thereby enhancing crop productivity and ecosystem services.

## 4. Materials and Methods

### 4.1. Experimental Fields and Plants

**Figure 6:**
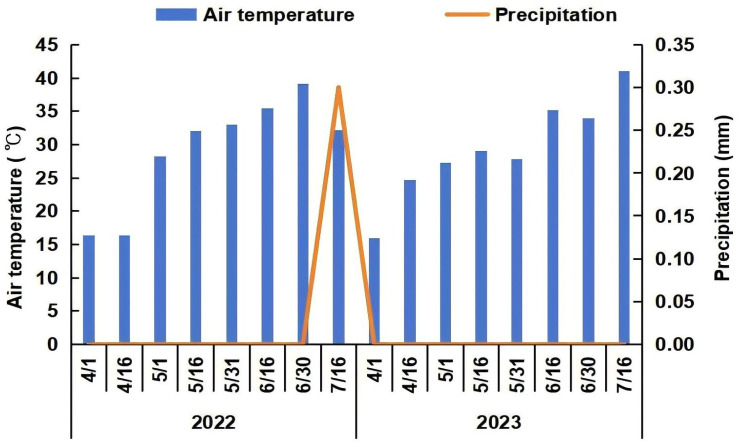
Precipitation and air temperature during the melon growth periods in 2022 and 2023.

The experiment was conducted at the experimental base of the Hami Melon Research Center in Yar Town, Gaochang District, Turpan, Xinjiang, China (42°13′ E, 89°15′ N). The soil type was sandy loam [50], and the basic physical and chemical properties of the 0–20 cm soil were as follows: organic matter 14 g/kg, total nitrogen 0.65 g/kg, total phosphorus 0.72 g/kg, total potassium 1.93 g/kg, hydrolyzable nitrogen 95.42 mg/kg, available phosphorus 20.35 mg/kg, and available potassium 272 mg/kg. Two treatments were established: conventional tillage (rotary tillage) and deep tillage. Conventional rotary tillage was performed using a rotary tiller to break up the topsoil to a depth of 15 cm. Deep tillage was performed by first using a rotary tiller and then using a moldboard plow to turn over the soil layer to a depth of 35 cm. Rotary and deep tillage were continuously carried out on the same land from 2022 to 2023. Samples were collected in 2023 for analysis. Each plot was 90 m^2^ (30 m × 3 m) in size with three replications. Two treatment plots were spaced 5 m apart. The melon cultivar used was “Nasimi”, provided by the Hami Melon Research Center of the Xinjiang Academy of Agricultural Sciences. The melons were sown in early April each year and harvested in mid-July. We trained a single vine per plant, retaining only one fruit for development. The row spacing was 3 m, the plant spacing was 0.4 m, and uniform field management was applied. The average daily air temperature and precipitation data during the growth period from 2022 to 2023 were obtained from Gaochang Meteorological Bureau, as shown in Figure 6.

### 4.2. Determination of Soil-Related Parameters

Soil samples were collected from 0–10 and 10–20 soil layers during the ripening period of melons in each treatment according to the “S” distribution method. Roots, weeds, and other impurities were removed using a 2 mm sieve. The soil samples were stored at 4 °C for enzyme activity analysis and dried at room temperature for physicochemical analysis. The 0–10 and 10–20 cm soil samples mentioned above were mixed, sieved in the same way, and stored at −80 °C for high-throughput sequencing, all with 3 biological replicates.

Soil pH was determined in a soil-to-water (1:2.5, *w*/*v*) mixture of dry soil and distilled water using a HACH HQ30d pH meter (BANTE, Shanghai, China). Total nitrogen (TN) content was determined using the Kjeldahl method, total phosphorus (TP) content was determined using spectrophotometry, total potassium (TK) content was determined using flame photometry, available phosphorus (AP) content was determined using the molybdenum antimony colorimetric method, and available potassium (AK) content was determined using flame photometry [51]. Soil sucrase activity was determined using the 3, 5-dinitrosalicylic acid colorimetric method, soil urease activity was determined using the indigo colorimetric method, and soil alkaline phosphatase activity was determined using the sodium diphenylphosphate colorimetric method [52,53,54]. Soil sucrase activity was determined using five microliters (5 mL) of phosphate buffer (pH 5.5) and five drops of toluene at 37 °C for 24 h. Soil urease activity was measured using a pH 6.7 citrate acid buffer solution at 37 °C or 24 h. Soil alkaline phosphatase activity was determined by the addition of 10 mL disodium phenyl phosphate solution as a substrate and phenol as a product after incubation at 37 °C for 24 h.

Then, 0.25 g of each soil sample from the 0–20 cm soil layer was weighed, and soil total microbial genomic DNA was extracted using the Power Soil^®^DNA Isolation kit (MOBIO, Carlsbad, CA, USA). Three biological replicates were performed for each treatment (for a total of 6 samples). Primers 338F (5′-ACTCCTACGGGAGCCAGCAG-3′) and 806R (5′-GGACTACHVGGGTWTCTAAT-3′) were used to amplify the V3–V4 region of the bacterial 16S rRNA gene. The ITS region of the fungi was amplified with primers ITS1F (5′-CTTGTCATTTAGAGAAGTAA-3′) and ITS2R (5′-GCTGCGTTTCTTCATCGATGC-3′). The size of the DNA fragments was checked by electrophoresis on a 1% agarose gel, and the DNA samples were stored at −20 °C for further analysis. The PCR products were amplified three times, electrophoresed on a 2% agarose gel, and purified using the Agencourt AMPure XP nucleic acid purification Kit (Beckman Coulter, Bria, CA, USA). After quality confirmation, the DNA samples were sent to Beijing Biomarker Technologies Co., Ltd. (Beijing, China) for Illumina Mi SeqTM sequencing.

### 4.3. Root Morphology and Yield Determination

Three well-grown melons with similar growth patterns were selected for each treatment, and intact root samples were carefully cut. After sampling, the soil–root mixture was placed in a 100-mesh nylon net and soaked in water for 0.5 h. Then, it was rinsed with tap water to remove impurities. The roots were then layered flat on the glass plate of the root scanner, scanned in grayscale mode using the root scanner, and saved as pixel 200 api, grayscale TIF files. The WinRHIZO software (Regent Instruments Inc., Quebec City, QC, Canada) was used to analyze and determine the total root length, root surface area, root volume, mean root diameter, root tip number, and fractal dimension of melon roots under different tillage measures at the maturity stage.

When melons were harvested, 10 melons were randomly picked from each plot, and the weight of a single melon was calculated and converted to yield per unit area based on planting density [55].

### 4.4. Data Analysis and Processing

Data analysis and mapping were completed using Graph Pad Prism (V8.0.1). The data were presented as the mean ± Standard Error (SE). IBMSPSS Statistics 20.0 (Chicago, IL, USA) was used for an independent sample *t*-test. Statistical analysis of OTU richness via Good’s coverage, Chao1, and Shannon’s index was performed with Mothur (v1.22.2). Redundancy analysis (RDA) of microbial community structure with environmental factors was executed by the R (v3.6.0) package “vegan”. Venn diagrams were constructed using Origin 2021.

## 5. Conclusions

This study investigated the soil environment and crop growth in response to tillage practices. Compared to conventional rotary tillage, deep tillage (DT) significantly elevated the total phosphorus and readily available phosphorus contents in the 0–20 cm soil layer. High-throughput sequencing results revealed that fungi exhibited higher richness (ACE and Chao1 indices) and diversity (Shannon index) compared to bacteria under different tillage practices. Root growth parameters indicated that DT treatment markedly enhanced melon root length, root surface area, root volume, mean root diameter, and root system architecture parameters. Melon yield was also significantly increased under DT treatment. DT treatment optimized root distribution in the soil and promoted melon root development and yield by improving soil nutrient conditions and influencing soil fungal community characteristics. The findings offer valuable theoretical insights for the implementation of effective tillage practices in open-field melon cultivation.

## Figures and Tables

**Table 1:** Changes in soil physical and chemical properties under different tillage practices.

Soil Layer (cm)	Tillage Modes	pH	TP (g/kg)	TK (g/kg)	TN (g/kg)	AP (mg/kg)	AK (mg/kg)
0–10	RT	7.54 ± 0.18 a	0.53 ± 0.02 b	11.86 ± 0.82 b	0.79 ± 0.06 b	39.17 ± 1.31 b	317.86 ± 1.21 b
	DT	6.49 ± 0.12 b	0.97 ± 0.04 a	14.27 ± 0.60 a	0.92 ± 0.03 a	50.01 ± 1.14 a	388.07 ± 2.74 a
10–20	RT	6.46 ± 0.17 b	0.47 ± 0.04 b	13.07 ± 0.44 a	0.77 ± 0.04 a	30.35 ± 1.42 b	334.61 ± 0.66 a
	DT	6.87 ± 0.16 a	1.18 ± 0.10 a	12.84 ± 0.93 a	0.80 ± 0.02 a	86.90 ± 2.60 a	335.54 ± 4.05 a

Note: Different letters in RT (0–10) vs. DT (0–10) and RT (10–20) vs. DT (10–20) show significant difference (*p* < 0.05) according to an independent sample *t*-test. RT, rotary tillage; DT, deep tillage; TN, total nitrogen; TP, total phosphorus; TK, total potassium; AP, available phosphorus; AK, available potassium.

**Table 2:** Analysis of soil bacteria and fungi alpha diversity indices.

Tillage Modes	Fungi			Bacteria		
	ACE Index	Chao1 Index	Shannon Index	ACE Index	Chao1 Index	Shannon Index
RT	330.41 ± 12.45 b	329.76 ± 12.55 b	4.66 ± 0.12 a	2250.37 ± 5.59 a	2241.84 ± 51.06 a	9.71 ± 0.21 a
DT	516.67 ± 22.13 a	515.87 ± 23.61 a	5.39 ± 0.41 a	2237.59 ± 15.08 a	2330.11 ± 155.52 a	9.88 ± 0.24 a

Note: Different letters in the same column show significant difference (*p* < 0.05) according to an independent sample *t*-test. RT, rotary tillage; DT, deep tillage.

**Table 3:** Root traits of melon under different tillage treatments.

Tillage Modes	Length (cm)	Surface Area (cm^2^)	Volume (cm^3^)	Mean Diameter (mm)	Root Tip Number	Fractal Dimension
RT	348.50 ± 1.57 b	173.40 ± 1.37 b	15.18 ± 1.98 b	1.10 ± 0.02 b	353.75 ± 1.22 b	1.59 ± 0.02 b
DT	513.77 ± 2.20 a	271.71 ± 1.42 a	24.11 ± 1.57 a	1.40 ± 0.02 a	438.25 ± 2.12 a	1.62 ± 0.01 a

Note: Different letters in the same column show significant difference (*p* < 0.05) according to an independent sample *t*-test. RT, rotary tillage; DT, deep tillage.

**Table 4:** Yield of melon under different tillage treatments.

Tillage Modes	Weight per Fruit (kg)	Vertical Diameter (cm)	Transverse Diameter (cm)	Fruit Shape Index	Yield (t/hm^2^)
RT	0.99 ± 0.20 b	14.89 ± 0.60 a	10.70 ± 0.32 b	1.39 ± 0.06 a	15.37 ± 1.25 b
DT	1.53 ± 0.09 a	17.50 ± 0.97 a	13.40 ± 0.15 a	1.30 ± 0.07 a	27.59 ± 1.69 a

Note: Different letters in the same column show significant difference (*p* < 0.05) according to an independent sample *t*-test. RT, rotary tillage; DT, deep tillage.

## Data Availability

Data are contained within the article.

## Supplementary Materials

The following supporting information can be downloaded at https://www.mdpi.com/article/10.3390/plants13182611/s1: Figure S1: Analysis of root phenotype of melon under different tillage practices.
