# Supplementary figures and images for: Effects of Rotary and Deep Tillage on Soil Environment and Melon Root Development
Source: Plants (Basel). 2024 Sep 19;13(18):2611. doi: 10.3390/plants13182611 (PMC11435252; doi:10.3390/plants13182611)

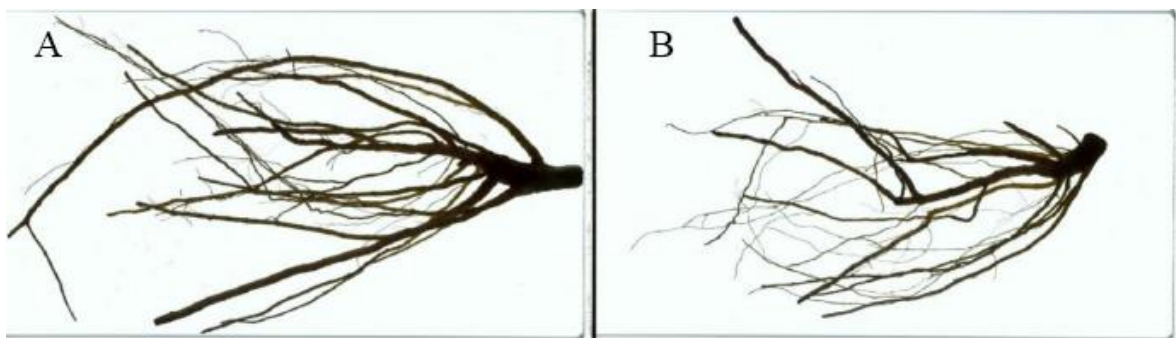

**Figure S1.** Analysis of root phenotype of melon under different tillage practices.

Supplement: Supplementary file 1 [file plants-13-02611-s001.zip › plants-3188644-supplementary.pdf]
